# Germline pathogenic variants associated with triple-negative breast cancer in US Hispanic and Guatemalan women using hospital and community-based recruitment strategies

**DOI:** 10.1007/s10549-024-07300-2

**Published:** 2024-03-23

**Authors:** Jesica M Godinez Paredes, Isabel Rodriguez, Megan Ren, Anali Orozco, Jeremy Ortiz, Anaseidy Albanez, Catherine Jones, Zeina Nahleh, Lilian Barreda, Lisa Garland, Edmundo Torres-Gonzalez, Dongjing Wu, Wen Luo, Jia Liu, Victor Argueta, Roberto Orozco, Eduardo Gharzouzi, Michael Dean

**Affiliations:** 1 Division of Cancer Epidemiology and Genetics, National Cancer Institute, NIH, Gaithersburg, MD USA; 2 Instituto Cancerologia, Guatemala City, Guatemala; 3 Texas Tech University Health Sciences Center, Lubbock, TX USA; 4 Cleveland Clinic Florida, Weston, FL USA; 5 Hospital General San Juan de Dios, Guatemala City, Guatemala; 6 Cancer Genetics Research Laboratory, Division of Cancer Epidemiology and Genetics, Frederick National Laboratory for Cancer Research, Gaithersburg, MD USA; 7 Integra Cancer Center, Guatemala City, Guatemala; 8 National Cancer Institute, 9615 Medical Center Drive, Rm 3130, Rockville, MD 20850 USA

**Keywords:** Early age, Pathogenic variants, Latin America, Health disparities, Global health, Socioeconomic, Mammography, Hereditary, Breast cancer, Triple-negative

## Abstract

**Purpose:**

Recruit and sequence breast cancer subjects in Guatemalan and US Hispanic populations. Identify optimum strategies to recruit Latin American and Hispanic women into genetic studies of breast cancer.

**Methods:**

We used targeted gene sequencing to identify pathogenic variants in 19 familial breast cancer susceptibility genes in DNA from unselected Hispanic breast cancer cases in the US and Guatemala.

Recruitment across the US was achieved through community-based strategies. In addition, we obtained patients receiving cancer treatment at major hospitals in Texas and Guatemala.

**Results:**

We recruited 287 Hispanic US women, 38 (13%) from community-based and 249 (87%) from hospital-based strategies. In addition, we ascertained 801 Guatemalan women using hospital-based recruitment. In our experience, a hospital-based approach was more efficient than community-based recruitment. In this study, we sequenced 103 US and 137 Guatemalan women and found 11 and 10 pathogenic variants, respectively. The most frequently mutated genes were *BRCA1*, *BRCA2*, *CHEK2*, and *ATM*. In addition, an analysis of 287 US Hispanic patients with pathology reports showed a significantly higher percentage of triple-negative disease in patients with pathogenic *variants* (41% vs. 15%). Finally, an analysis of mammography usage in 801 Guatemalan patients found reduced screening in women with a lower socioeconomic status (*p* < 0.001).

**Conclusion:**

Guatemalan and US Hispanic women have rates of hereditary breast cancer pathogenic variants similar to other populations and are more likely to have early age at diagnosis, a family history, and a more aggressive disease. Patient recruitment was higher using hospital-based versus community enrollment. This data supports genetic testing in breast cancer patients to reduce breast cancer mortality in Hispanic women.

**Supplementary Information:**

The online version contains supplementary material available at 10.1007/s10549-024-07300-2.

## Introduction

Breast cancer is the most prevalent cancer among women worldwide (1), with a mortality rate of 30% in 2020. Breast cancer incidence is higher in countries with higher socioeconomic status, but mortality rates are lower. In contrast, in low-and-middle-income countries (LMIC), breast cancer has a lower prevalence rate but a higher mortality rate among women (2017 GLOBOCAN). This disparity is likely due to treatment availability and early detection in higher-income countries. In 2020 breast cancer accounted for 24% of cancer cases among women in Guatemala [1]. Whereas in women in the United States (US), in 2019, 30% of newly diagnosed cancer cases were breast cancer [2].

Most genetic and epidemiological studies in the US have focused on populations of European descent [3]. Although there are programs centered on Hispanic populations, there are obstacles to the recruitment of minority women in research, such as community engagement, language, and cultural barriers [4]. Latin American and US Hispanic women are underrepresented in genetic breast cancer studies. Failure to detect *BRCA1* and *BRCA2* pathogenic variants can delay diagnosis and treatment. Increased availability and affordability of next-generation sequencing and annotation of genetic variants would aid in identifying inherited breast cancerpathogenic variantsamong Hispanic populations and reduce mortality. This study aims to analyze high penetrance breast cancer genes with pathogenic pathogenic variants in Hispanic women in US and Guatemalan women. Furthermore, we highlight the importance of patient recruitment strategies to increase the Hispanic women’s participation in genetic studies.

## Methods

### Patient recruitment in the US

Community recruitment across the US was conducted from July 2011 to August 2016. For community recruitment, we designed and received IRB approval for a protocol allowing patient ascertainment through the internet, by phone or in person (NCT0151900). The study was published online in ClinicalTrials.gov and advertised on social media. We also recruited patients at community centers, such as Nueva Vida, Baltimore and Richmond (https://www.nueva-vida.org/), the Avon Breast Cancer Walk, Washington, DC (https://www.avon.com/breast-cancer-crusade), and the Oklahoma Latino Community Development Agency. In addition, we sent letters to oncologists in predominantly Hispanic areas in Southern Florida., sending letters to oncologists in personal contacts with Hispanic cancer support and business groups, and through charities.

In Texas, we recruited subjects exclusively from the Texas Tech University Health Sciences Centers at Lubbock and El Paso. We invited women receiving care for current or previous breast cancer diagnoses to participate in the study. All US and Guatemala patients were administered a questionnaire in Spanish or English. The questionnaire for participants included age of diagnosis, demographics, reproductive history, socioeconomic status, and relevant family history of breast cancer. The questionnaire was identical for Guatemalan and US subjects (Online Appendix A).

### Patient recruitment in Guatemala

In Guatemala, all recruitment occurred directly within major medical centers. In Guatemala City, we recruited patients from the Hospital General San Juan de Dios (HGSJDD) and the Instituto Nacional de Cancerologia (INCAN). Both Hospitals serve the entire Guatemalan population, with patients referred by primary care physicians. Most breast cancer patients treated at INCAN were older than 40 (84%) and lived in the capital city, indicating a higher SES. Furthermore, younger patients came from regions west of Guatemala City (22%). The questionnaire for Guatemalan patients included age at the time of diagnosis, demographics, reproductive history, cooking on a wood stove (a measure of SES), and family history of breast cancer.

### IRB approval and patient consent

In Guatemala this study was conducted at the Hospital General San Juan de Dios (HGSJDD) and the Instituto de Cancerología (INCAN) in Guatemala City. The Research Ethical Committees of each institution approved the protocol, and the study was determined exempt from institutional review board (IRB) approval by the NIH Office of Human Studies Research. Women attending either of these hospitals for their breast cancer diagnostic biopsies were invited to participate and gave written informed consent. Two 5 ml tubes of blood were collected and frozen at − 20 °C as well as a tumor biopsy stored in 0.5 ml of RNAlater solution at − 20 °C. Trained interviewers administered an approved questionnaire including reproductive history, family history of cancer, and socioeconomic data.

In the U.S IRB protocol was approved by the NCI IRB and is listed in clinical trials.gov (https://www.clinicaltrials.gov/ct2/show/NCT01251900) (NCT01251900).

### Sequencing

We used targeted sequencing to identify pathogenic variants in blood DNA from unselected Hispanic breast cancer cases from community recruitment and from two hospitals each in Texas and Guatemala. A total of 137 blood samples from INCAN, HGSJDD, and 96 blood samples from Texas (El Paso and Lubbock) were sequenced on the NOVASeq from Illumina with the Paired-end 200 bp strategy [5]. Briefly, blood DNA (200 ng) were used to produce an adapter-ligated library the Kapa HyperPlus Kit (Roche, Indianapolis, IN) using xGen Dual Index UMI Adapters (IDT, Coralville, IA) according to Kapa-provided protocol. The resulting post-capture enriched multiplexed sequencing libraries were loaded on a NovaSeq 6000 (Illumina, San Diego, CA) and paired-end sequencing was performed using read lengths of 2 × 150 bp to an average coverage of 50×.

### Variant classification

We analyzed protein truncating variants in the genes *BRCA1, BRCA2, PALB2, CHEK2,* and *ATM*, as well as pathogenic missense variants in *TP53, BARD1, RAD51C,* and *RAD51D* [6]. All genes are listed in the Supplemental Table 1.

We annotated variants using SNPNexus for targeted breast cancer genes. We performed manual validation using Integrative Genomics Viewer (IGV) (https://igv.org/app). Then placed the variants into three categories pathogenic, VUS, and benign. Pathogenic variants were further confirmed using ClinVar (https://www.ncbi.nlm.nih.gov/clinvar/) and Varsome (https://varsome.com/).

### Breast cancer subtypes in US Hispanic women

Hormone receptor data was only available in the US Hispanic subjects. We characterized the four breast cancer subtypes as follows: (1) luminal A as estrogen receptor (ER) positive, progesterone receptor (PR) positive, HER2 receptor-negative, and low Ki 67. (2) Luminal B subtype ER positive, PR positive, HER2± , and high Ki67 count. (3) HER2+ subtype is ER negative, PR negative, and HER2 positive regardless of Ki67 count. (4) Triple-negative breast cancer is ER, PR, and HER2 receptor-negative. We combined ER and PR positive, HER2 negative tumors without Ki67 data into a luminal A/B group [7].

### Mammography screening

Self-reported mammography screening was available for both Guatemala and US Hispanic women. We calculated active mammography screening for patients over the age of 40. We determined the difference between the age of diagnosis and age at the first mammogram. If the difference was two years or greater, we classified the subject as receiving active screening. Patients with a first mammogram less than two years before diagnosis were classified as unscreened.

### Socioeconomic status (SES)

We used self-reported income brackets in the US to estimate socioeconomic status (SES). In Guatemala, SES is difficult to determine directly due to the lack of job security. As a proxy, we used cookstove type as an indicator of SES due to the known association between wood-burning stoves and poverty in Guatemala, particularly among indigenous Mayans (10.1186/ISRCTN29007942, guatemalastoveproject.org/). We confirmed that wood cookstove use is associated with Indigenous American ancestry (Supplemental Fig. 1).

### Statistics

We used a two-proportion Z-test (two-tailed) to assess the difference in the percentage of patients with a family history of breast cancer, contraception use, and parity. Second, we used an unpaired t-test with Welch’s correction to ascertain the relationship between breast cancer subtypes in US patients with pathogenic and non-pathogenic variants. Third, a Chi-squared test with Yates correction examined the relationship between socioeconomic status (cookstove type) and mammogram screen usage. Finally, Fisher’s exact test assessed the relationship between Guatemalan women’s cookstove use and Indigenous American ancestry. In all calculations, a p-value of 0.05 or less was deemed significant.

## Results

### Patient recruitment

**Fig. 1:**
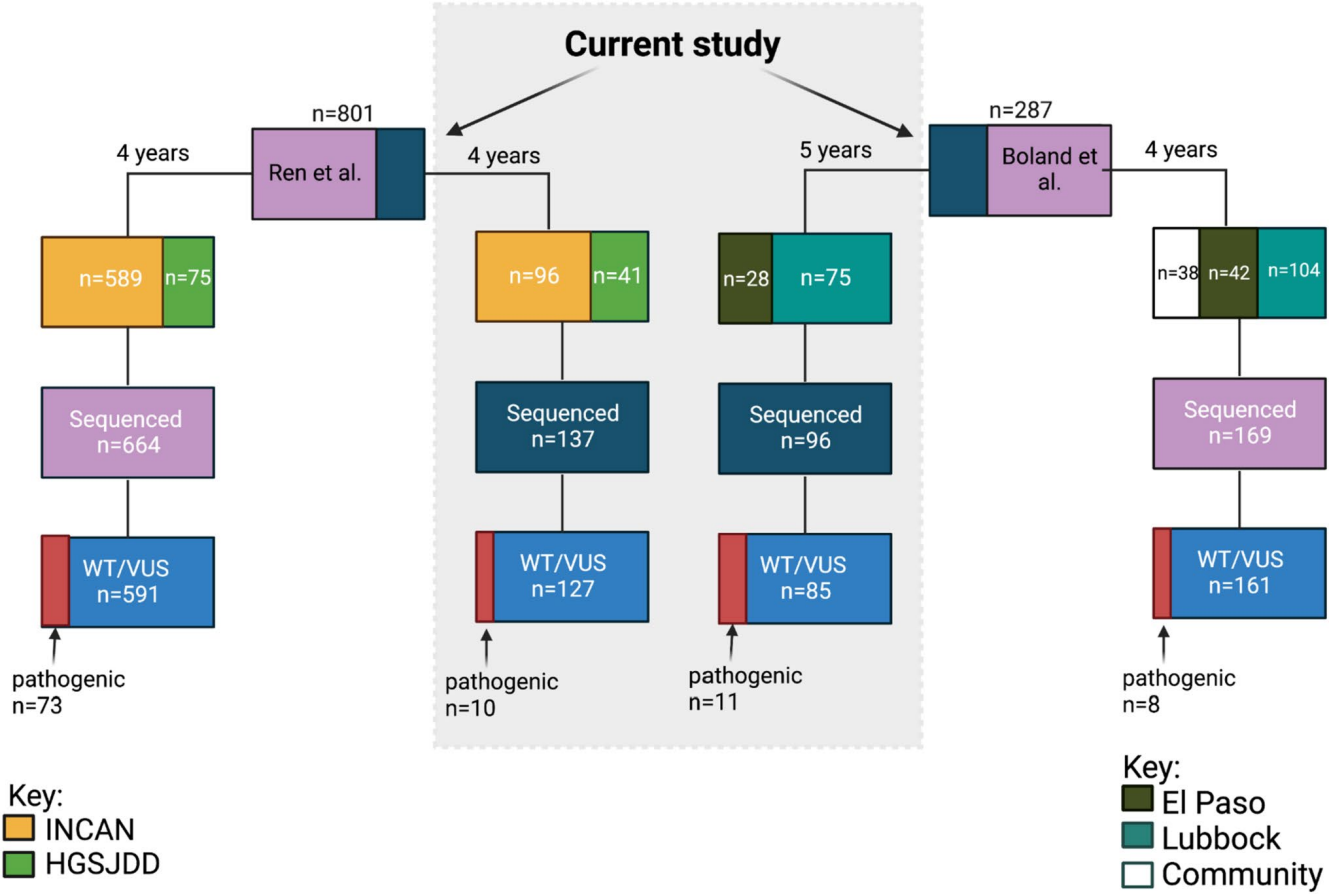
Recruitment of US Hispanic and Guatemalan women. We show the patient distribution of subjects in Guatemala and the US. There was a total enrollment of 801 unselected Guatemalan women through hospital-based recruitment over 5.25 years. Enrollment in the US occurred over 9 years with 287 unselected women, 38 from community recruitment, and 249 from a hospital-based approach. We report the US and Guatemalan patients in this study within the gray box. Partial data on additional subjects from the two cohorts have been previously published [5, 8]

To analyze the characteristics of breast cancer in Hispanic patients in the US and Guatemala we recruited women through community- and hospital-based strategies. Our current study ascertained 103 US Hispanic women from hospital-based recruitment in TTUHSC El Paso and Lubbock. We ascertained 137 women from the INCAN and HGSJDD hospitals in the Guatemala cohort. In Fig. 1, we indicate the number of women sequenced for germline pathogenic variants and report the total of each with and without pathogenic variants. In a previous study in the US, we described *BRCA1* and *BRCA2* pathogenic variants in 184 women [8]. Overall, in the US, we recruited 38 (13%) women through community-based recruitment and 249 (87%) from two hospitals in Texas (Table 1). In addition, we recruited a total of 801 patients using hospital-based recruitment in Guatemala City; 137 from our current study and 664 from a previous study [5].

**Table 1:** Demographics of US and Guatemalan women

	Total Guatemala *n* = 801		Total US n = 287		*p*-value
	Mean (IQR)	Range	Mean (IQR)	Range	
Age at diagnosis	49 (41–61)	19–93	52 (43–60)	23–81	0.57
Age at first mammogram	47 (40–59)	15–91	40 (35–45)	17–75	< 0.0001
Age at menarche	13 (12–14)	9–24	13 (11–14)	8–18	< 0.0001
Age at 1st pregnancies	21 (18–25)	14–43	21 (18–24)	13–42	0.37
Number of pregnancies	3.0 (2.0–5.0)	0–19	3.0 (2.0–4.0)	0–10	< 0.0001
Number of children	3.0 (2.0–4.0)	0–19	3.0 (2.0–4.0)	0–8	0.0004
Breast feeding	90%		50%		< 0.0001
Family history	14%		40%		< 0.0001

Analysis of patient demographics and risk factors known to be associated with the development of breast cancer. We report mean, interquartile ranges (IQR), and p-values for all Guatemala and US women*.* The family history reported is solely for family members with breast cancer.

### Demographics

In breast cancer, age is one of the most significant risk factors, as the development of the disease increases after the fourth decade of life [2]. The mean age at diagnosis for Guatemalan women was 49 years old, and for US Hispanic women 52 years old, with no significant difference between these two groups. Early age at menarche increases a woman’s risk of developing breast cancer due to earlier exposure to estrogen [9]. Guatemalan and US Hispanic women had the same mean age of menarche, 13 years old. However, the *mean age* in Guatemala was 13.2, and in the US was 12.6 (*p* < 0.0001) (Table 1). Decreased estrogen exposure can reduce a woman’s risk of developing breast cancer, and reproductive factors that decrease risk include higher parity, lower age at first birth, and breastfeeding [9]. Both groups’ mean age at first pregnancy was 21, with no differences between Guatemalan and US women. The mean of live births in Guatemala, 3.3 was significantly higher than in US women, 2.8 (*p* = 0.0004). Early diagnosis of breast cancer can ultimately result in an improved prognosis and reduced incidence of metastatic cancer. Currently, the US guidelines recommend mammography screening for women at 50 years old, and women with a family history of breast cancer are recommended mammography screening at 40 years old [10]. In our analysis, the mean age of Guatemalan women at their first mammogram was 47 years old, significantly higher (*p* < 0.0001) than US Hispanic women with a mean age of 40. Therefore, our data suggest that US Hispanic women, in our study, received mammography screening consistent with current guidelines.

### Analysis of pathogenic variants

We previously published pathogenic variants in 73 out of 664 Guatemalan patients and 10 out of 96 patients from the US Latina population. In this study we sequenced an additional 137 Guatemalan breast cancer cases and identified 10 pathogenic variants in high penetrance genes (Table 2**)**. We identified three additional cases of the c.212 + 1G > A founder mutation in *BRCA1*. Furthermore, we present current data on 96 additional US Hispanic patients, identifying 11 more pathogenic variants in high and moderate penetrance genes (Table 2**)**. We identified a carrier of the c.68_69delAG Ashkenazi Jewish founder mutation in one patient from Texas, who had an early age diagnosis (44 years) and a family history of breast cancer. In the US combined data set, cases with pathogenic variants had a significantly earlier age at diagnosis (41 vs. 52 years, *p* < 0.0001) and were more likely to have a diagnosis before menopause. In addition, a higher percentage of mutation carriers had a relative with breast cancer (39% vs. 67%, *p* < 0.01).

**Table 2:** Pathogenic mutations include cDNA position, protein alteration, gene, and pathological status

Study	cDNA name	Protein name	Penetrance	Gene	Chromosome	Age	COB	Family history	Recurrent mutation	Subtype status	Metastasis	Primary diagnosis
United States	c.68_69delAG	p.Glu23fs	High	BRCA1	chr17	44	United States	Yes	Yes	TNBC	No	IDC—invasive ductal carcinoma
United States	c.3675C > A	p.Cys1225*	High	BRCA1	chr17	44	Mexico	Yes		TNBC	No	IDC—invasive ductal carcinoma
United States	c.2433delC	p.Lys812fs	High	BRCA1	chr17	34	United States	Yes		TNBC	Metastatic	IPC—invasive papillary carcinoma
Guatemala	c.3454_3455insC	p.Asp1152fs	High	BRCA1	chr17	47	Guatemala	No		Not determined	Not determined	Not determined
Guatemala	c.212 + 1G > A		High	BRCA1	chr17	37	Guatemala	Yes	Yes	Not determined	Not determined	IDC—invasive ductal carcinoma
Guatemala	c.212 + 1G > A		High	BRCA1	chr17	38	Guatemala	Yes	Yes	Not determined	Not determined	IDC—invasive ductal carcinoma
Guatemala	c.212 + 1G > A		High	BRCA1	chr17	66	Guatemala	Yes	Yes	Not determined	Not determined	IDC—invasive ductal carcinoma
United States	c.3264dupT	p.Gln1089fs	High	BRCA2	chr13	45	United States	Yes	Yes	A/B	Not determined	DCIS—ductal carcinoma in situ
United States	c.2808_2811delACAA	p.Ala938fs	High	BRCA2	chr13	40	Spain	Yes		B	Not determined	IDC—invasive ductal carcinoma
United States	c.3264dupT	p.Gln1089fs	High	BRCA2	chr13	44	Mexico	Yes	Yes	TNBC	Not determined	DCIS—ductal carcinoma in situ
Guatemala	c.5308delT	p.Ser1770fs	High	BRCA2	chr13	25	Guatemala	No		Not determined	Not determined	Not determined
Guatemala	c.6531_6534delTCAT	p.Ile2177fs	High	BRCA2	chr13	54	Guatemala	No		Not determined	Not determined	IDC—invasive ductal carcinoma
Guatemala	c.9235delG	p.Val3079fs	High	BRCA2	chr13	57	Guatemala	Yes		Not determined	Not determined	Not determined
Guatemala	c.658_659delGT	p.Val220fs	High	BRCA2	chr13	40	Guatemala	No		Not determined	Not determined	IDC—invasive ductal carcinoma
Guatemala	c.8489G > A	p.Trp2830*	High	BRCA2	chr13	28	Guatemala	No		Not determined	Not determined	IDC—invasive ductal carcinoma
United States	c.417C > A	p.Tyr139*	High	CHEK2	chr22	40	Guatemala	Yes		Unknown	No	IDC—invasive ductal carcinoma
United States	c.433C > T	p.Arg145Trp	High	CHEK2	chr22	36	United States	unk		A/B	No	IMC—invasive mucinous carcinoma
United States	c.2921 + 1G > A		High	ATM	chr11	52	Mexico	No		TNBC	No	IDC—invasive ductal carcinoma
United States	c.8977C > T	p.Arg2993*	High	ATM	chr11	59	United States	Yes		B	Metastatic	IDC—invasive ductal carcinoma
Guatemala	c.731G > A	p.Gly244Asp	High	TP53	chr17	47	Guatemala	Yes		Not determined	Not determined	Not determined
United States	c.1240delA	p.Met414fs	Moderate	BARD1	chr2	51	United States	Yes		TNBC	No	IDC—invasive ductal carcinoma

Pathogenic mutation and the age of diagnosis, country of birth, pathology, and family history of breast cancer are shown. The most frequently mutated genes were the high penetrance genes BRCA1, BRCA2, CHECK2, and ATM

### Breast cancer subtypes

**Fig. 2:**
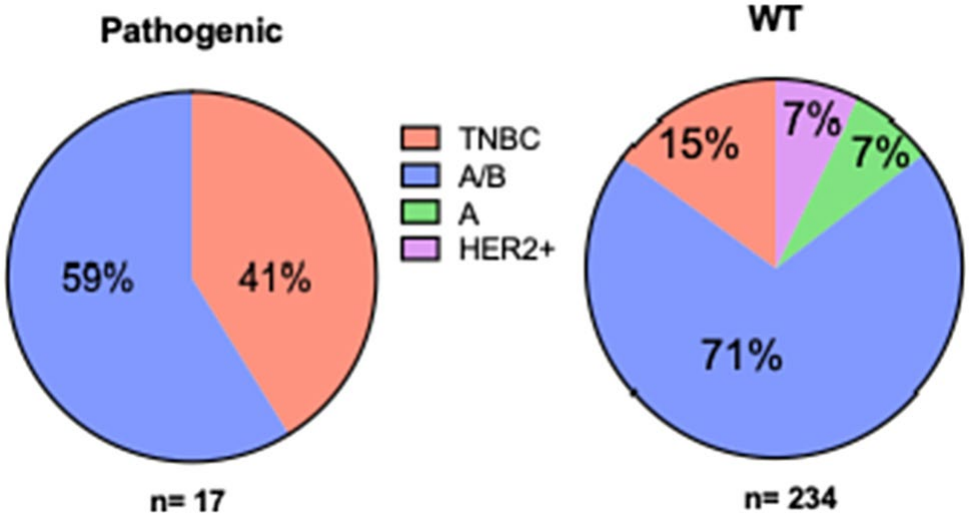
Breast cancer subtypes by mutation status. Analysis of breast cancer subtypes in 251 US patients with (*n* = 17) and without pathogenic variants (*n* = 234). Subtypes were luminal A (green), luminal A/B (luminal A or B) (blue), HER2 + subtype (pink), and triple-negative (TNBC) (red)

To determine if cases with a pathogenic mutation have a higher rate of triple-negative disease, we analyzed breast cancer subtypes in the entire US Hispanic population data set. Patients with pathogenic variants had a higher percentage of triple-negative disease (41% vs. 15%, *p* = 0.9), and there were no HER2 + cases in this group (Fig. 2). Therefore, US Hispanic women with pathogenic pathogenic variants have a higher frequency of aggressive breast cancer subtypes (TNBC) with poorer prognosis.

### Mammography screening

**Fig. 3:**
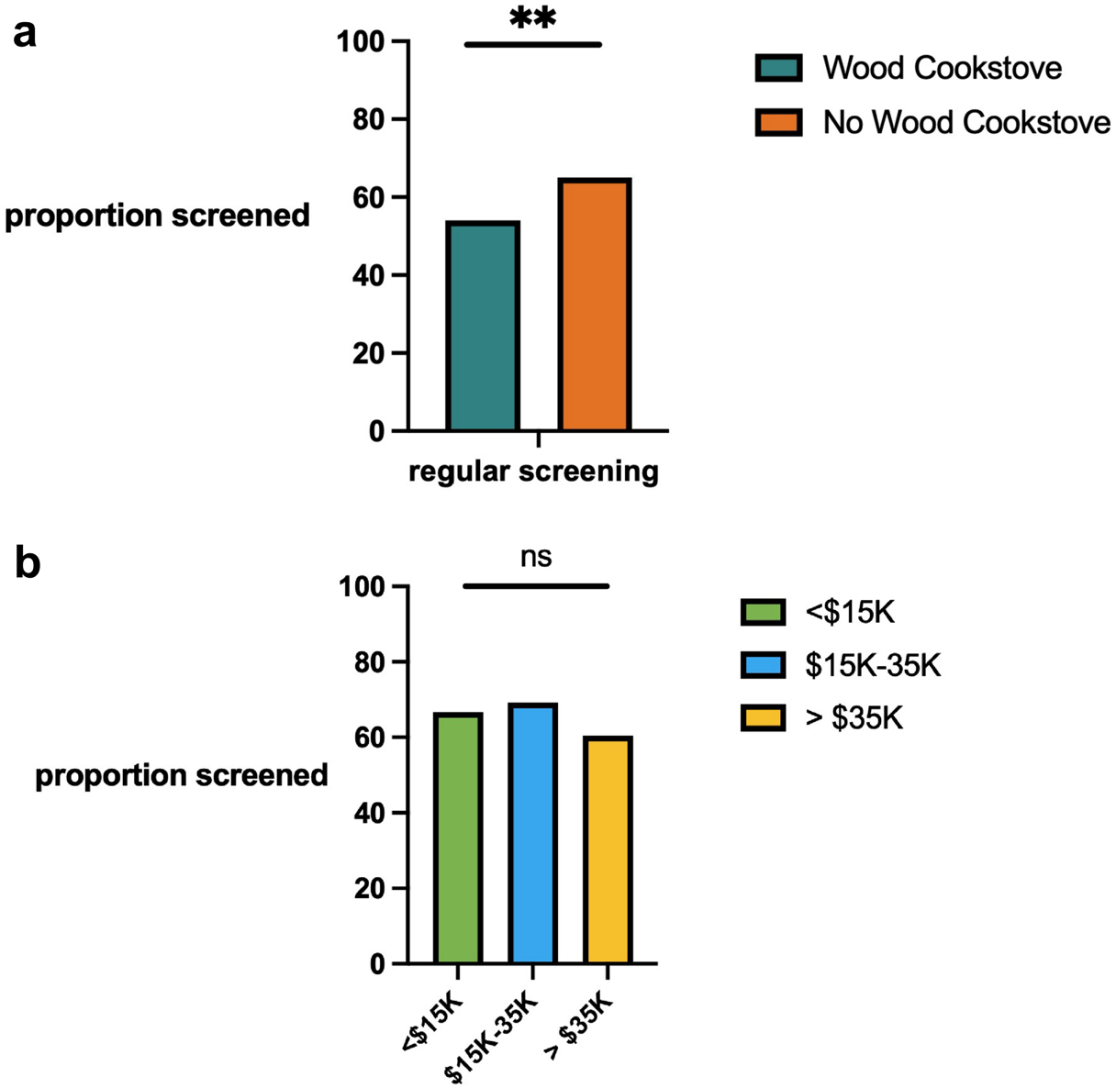
Proportion of women receiving mammography screening by socioeconomic status. **a** Regular mammogram screening was compared in women who do or do not use a wood cooking stove in Guatemala as a measure of SES. **b** Proportion of mammographic screening in US women’s by income group

The American Cancer Society measures breast cancer screening rate by the percentage of women 40 and older who had a mammogram in the past two years (American Cancer Society). We sought to identify a connection between mammogram use and socioeconomic status. Self-reported mammography data was available for 611 Guatemalan women over 40. In total, 362 women (59%) indicated that they had received a mammogram, but only 249 (41%) had regular screening in the two years preceding their diagnosis (Fig. 3a). Women in Guatemala did not report income; therefore, we used cooking with wood as a proxy for lower SES and for Indigenous American ancestry (Supplemental Fig. 1). We found that mammography usage was less frequent in women cooking with wood, indicating that this group received inadequate breast cancer screening (*p* < 0.0045).

For women in the US, self-reported household income was available. We aggregated household income into three groups: less than $15,000, $15,000 to $35,000, and greater than $35,000. Mammography usage was not significantly different between SES groups in the US (Fig. 3b), indicating that breast cancer screening is prevalent in US Texas women. The average household income in the US in 2021 was $70,784, whereas most women in our cohort were under the household median income. Therefore, despite the low socioeconomic status of the women in the US we studied, they are receiving regular mammography screenings.

### Metastasis in US women and SES

**Fig. 4:**
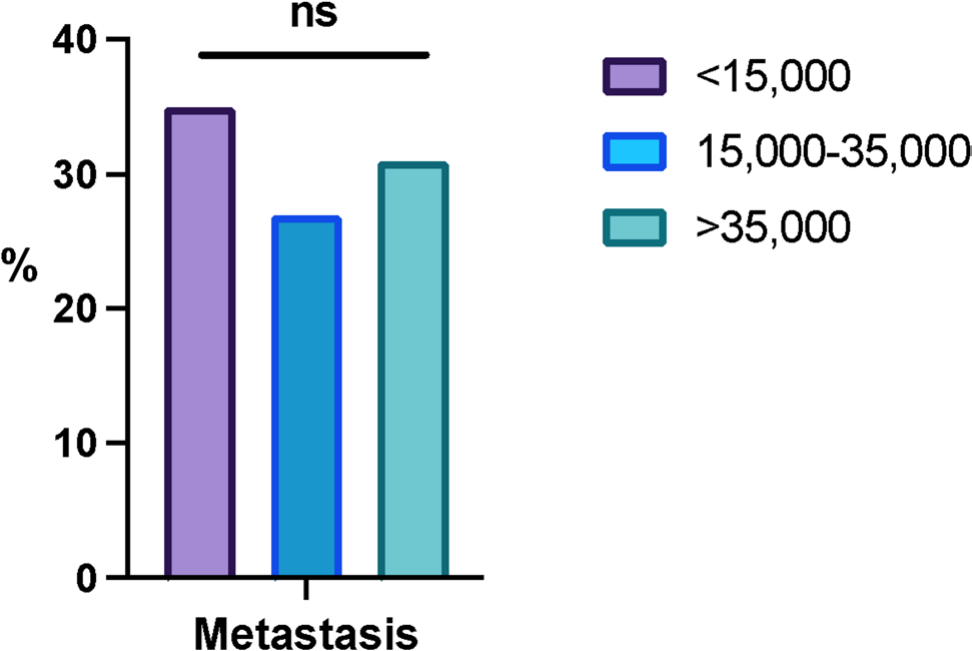
Metastatic breast cancer is similar among women in the US. We compared metastatic breast cancer rates and socioeconomic status. Three groups: less than $15,000, $15,000–$35,000, and greater than $35,000, were compared for the percentage of metastatic disease

We compared income groups to determine if the prevalence of metastatic breast cancer is related to SES in US women. Between the three income subgroups, there was no significant difference in the percentage of metastasis (Fig. 4). Therefore, women with an income less than $15,000 are being provided equal mammography screening as the other groups and are not developing a higher rate of metastatic disease, indicating there is no detectable health disparity for metastatic disease.

## Discussion

To address the underrepresentation of Latin American/Hispanic women in breast cancer genetic studies, we explored two strategies for patient recruitment: a community-based model and a hospital/cancer center-based model. Although we reached a broad audience, only 38 patients were enrolled by this approach. We observed that in-person presentations in Spanish to breast cancer patients and survivors resulted in high participation. However, the study needed full-time staff and resources to pursue this approach on a national level. Establishing the protocol in hospitals with sizable Hispanic patient populations in the US, such as Lubbock and El Paso, Texas, as well as an adult cancer hospital and a large general hospital in Guatemala, was far more effective. Although the study did not have funds for the Texas hospitals to pay for staff time, we still enrolled 179 subjects in 7.5 years in Lubbock and 70 subjects in 0.5 years in El Paso. In Guatemala, we funded staff to recruit, consent, administer questionnaires, collect, and ship samples, and this yielded 685 subjects in 5.25 years at INCAN and 116 subjects in 3.5 years at HGSJDD. We conclude that community-based recruitment can be effective, but to be successful requires in-person contact with patients and a dedicated bilingual staff. Hospital-based studies in areas with a substantial Hispanic population are also effective, if adequate staff is available.

Large patient studies analyzing the participation of minorities in genetic studies found the participation of Hispanic women was 73–87% [4]. The recruitment strategies for these studies included telephoning patients directly and providing transportation for biospecimen collection. Although these studies demonstrated effective community-based recruitment, the authors emphasized the need for substantial financial support for extensive genetic studies [4, 11].

### Genetic testing

Genetic testing is largely unavailable in most low- and middle-income countries like Guatemala, resulting in higher morbidity and mortality. In addition, genetic testing is underutilized in US Hispanic populations due to a lack of insurance and understanding the value of genetic testing. However, the increase of Latin American women in genetic studies improves health outcomes. Our data shows that 10% of Latin American unselected women have a germline pathogenic mutation. Furthermore, similar to other populations, Latin American mutation carriers have an earlier age of onset, triple-negative disease, and relevant family history.

Our current data on pathogenic variants extend results we previously published on these patient populations [5, 8], finding additional examples of the 212 + 1 G > A founder mutation in Guatemala [12]. We also identified one case from Texas carrying the c.68_69delAG/185delAG mutation. This mutation is prevalent among women of Ashkenazi-Jewish descent [13]. In previous studies, 185delAG was seen in specific Latin American and Mexican American populations as the most common mutation [14, 15]. The 185delAG carrier we identified had triple-negative breast cancer with metastasis, was diagnosed at 44, and had first- and second-degree relatives with breast cancer. Women with pathogenic variants can benefit from enhanced screening and prophylactic surgery to prevent breast and ovarian cancer. Therefore, enhanced use of genetic testing and counseling would result in earlier diagnosis and improve outcomes across Hispanic communities in the US and Latin America.

Genome-wide association studies (GWAS) of breast cancer have identified over 170 loci [16] associated with this disease. Interestingly, a GWAS study in 1,497 Latina women in the US identified a protective variant (rs140068132) on chromosome 6q25. This variant is found almost exclusively in people of Indigenous American ancestry. Furthermore, the variant is associated with lower mammographic density, reduced ER-negative cancer, and reduced overall breast cancer risk (16%). This study further emphasizes the importance of genetic studies in Latin American women [17].

### Triple-negative breast cancer

Breast cancer subtyping is critical to determining therapy and outcomes. For example, triple-negative breast cancer is associated with a higher rate of metastasis, lower response to therapy, and lower survival rates than other breast cancer subtypes [18, 19].

TNBC prevalence varies by ethnicity, with approximately 10% in women of European descent, 30% in African American women, and 10–20% in Hispanic women [20–22]. In our study, 17% of US Hispanic women had triple-negative breast cancer. Women with germline pathogenic variants had a significantly higher frequency (41%) of TNBC, than in women without (15%) (*p* = 0.01). *BRCA1* and to a lesser extent, *BRCA2* pathogenic variants were associated with TNBC, as seen in other populations [23].

### Limitations

The limitations of this study include the use of self-reported data in the questionnaire, and that information on treatment and outcomes was not available. In Guatemalan women, direct SES data were unavailable; therefore, we used cooking with wood to ascertain women with a low SES and gas stove cooking for higher SES. In addition, the sample size of the community-recruited US Hispanic women was modest.

## Summary

There is limited representation of Latin American women in genetic studies and extensive epidemiology studies rely on hospital-based databases. Hospital-based recruitment proved more productive due to the continuity of communication and recordkeeping, and community recruitment requires substantial financial support.

In both the US and Guatemala, women with pathogenic variants have a significantly younger age of diagnosis. In addition, they are more likely to have a first- or second-degree relative with breast cancer. Also, women with a pathogenic variant were more likely to develop triple-negative disease. Our results emphasize the importance of the availability of genetic testing for all women with breast cancer.

## Supplementary Information

Below is the link to the electronic supplementary material.

Supplementary file1 (PDF 467 KB)

## Data Availability

The datasets generated during and/or analyzed during the current study are available in the dbGAP repository, dbGaP Study Accession: phs002246.v1.p1.
