# Supplementary material for: Germline pathogenic variants associated with triple-negative breast cancer in US Hispanic and Guatemalan women using hospital and community-based recruitment strategies
Source: Breast Cancer Res Treat. 2024 Mar 23;205(3):567–77. doi: 10.1007/s10549-024-07300-2 (PMC11101360; doi:10.1007/s10549-024-07300-2)
Supplement: Supplementary file 1 — Supplementary file1 (PDF 467 KB) [file 10549_2024_7300_MOESM1_ESM.pdf]

Supplemental materials

Breast Cancer Research and Treatment: Germline Mutations Associated with Triple Negative Breast Cancer in US Hispanic and Guatemalan Women using Hospital and Community-Based Recruitment Strategies, **Jesica Godinez Paredes[1], Isabel Rodriguez[1], Megan Ren[1], Anali Orozco[2], Jeremy Ortiz[2], Anaseidy Albanez[2], Catherine Jones[3], Zeina Nahleh[4], Lilian Barreda[5], Lisa Garland[6], Edmundo Torres Gonzalez[1], Dongjing Wu[6], Wen Luo[6], Jia Liu[6], Victor Argueta[5], Roberto Orozco[5], Eduardo Gharzouzi[7], Michael Dean (deanm@mail.nih.gov)**

Supplementary Figure 1. In Guatemalan women, SES could not be directly determined. Therefore, we used cooking with wood to ascertain a low SES in Guatemalan women and gas stove cooking for a higher SES. Women that cook with a wood stove had higher Indigenous American ancestry (>0.5).

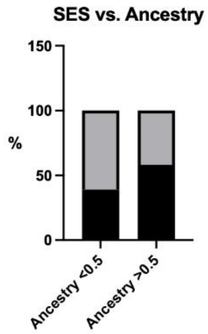

Supplementary Figure 2. The entire data set of patient demographics. a) Combining the data for all US Hispanic women with pathogenic (n=18) and wild-type (n=249), we found significant age of onset (p<0.0001) and family history (p<0.0001). **b)** Guatemala age of onset (p<0.0001), and family history (p<0.0001).

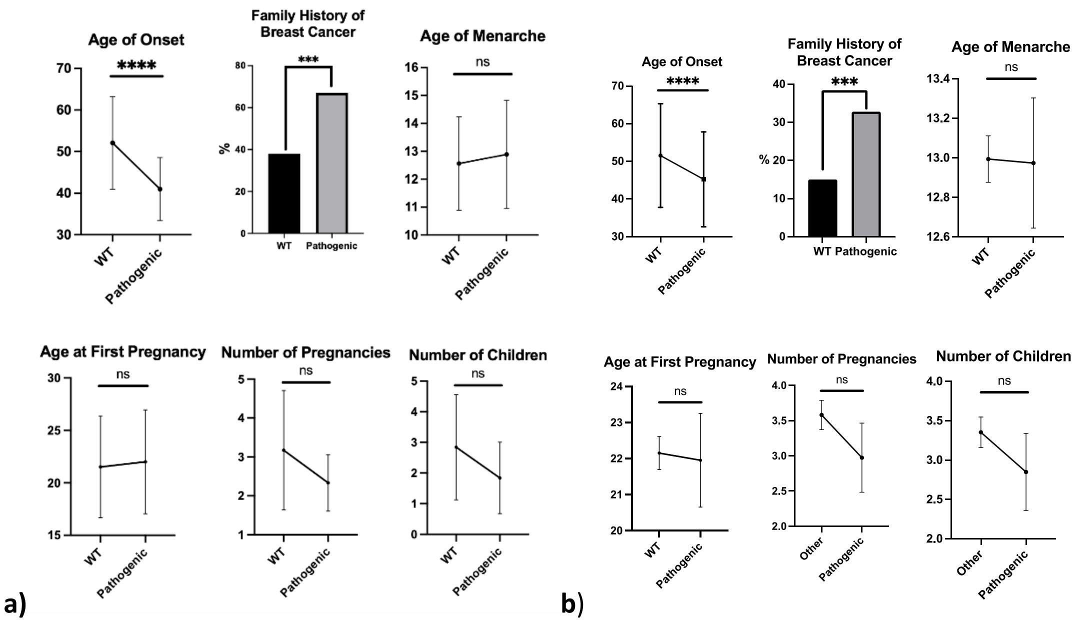
Age of Onset
****
WT
Pathogenic
Family History of Breast Cancer
***
%
WT
Pathogenic
Age of Menarche
ns
WT
Pathogenic
Age at First Pregnancy
ns
WT
Pathogenic
Number of Pregnancies
ns
WT
Pathogenic
Number of Children
ns
WT
Pathogenic
a)
b)
Age of Onset
****
WT
Pathogenic
Family History of Breast Cancer
***
%
WT Pathogenic
Age of Menarche
ns
WT
Pathogenic
Age at First Pregnancy
ns
WT
Pathogenic
Number of Pregnancies
ns
Other
Pathogenic
Number of Children
ns
Other
Pathogenic

Supplemental Figure 3. Percentage of mutations by gene.

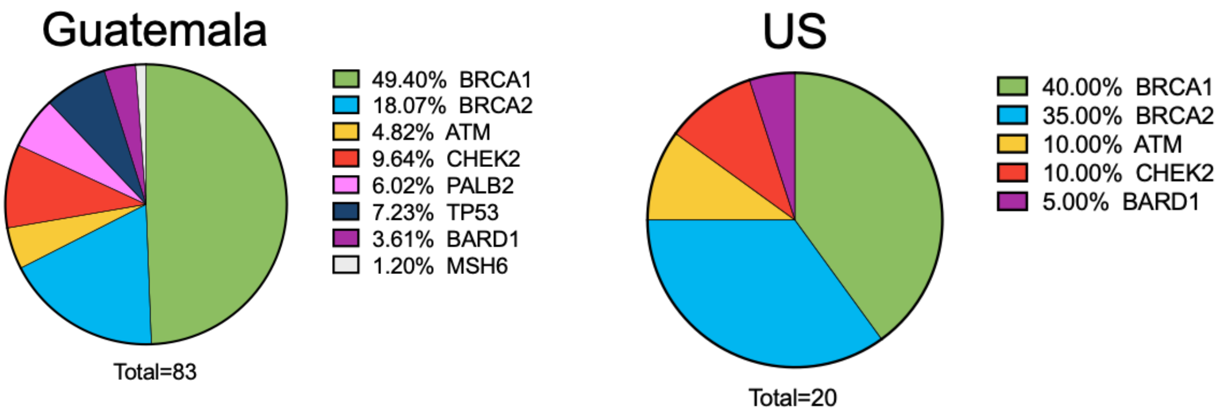

Supplemental Table 1. Classification of genes used in analysis.

Protein truncating variants			
**High**		**Moderate**	
ATM	*BARD1*	*BRIP1*	*MSH6*
BRCA1	*PTEN*	*CDH1*	*MUTYH*
BRCA2	*RAD51C*	*FANCC*	*PMS2*
CHEK2	*RAD51D*	*FANCM*	*RAD50*
PALB2	*TP53*	*MSH2*	

Supplemental Table 2. Analysis of related patients in the Guatemalan study. We analyzed a subset of patients by SNP array and identified 8 related patients with a first or second-degree relative positive for breast cancer. Two of the pairs of relatives shared the same pathogenic mutation. (**Supplemental Table 3**).

Patient 1	Mutation		FamHistory	Patient 2	Mutation		FamHistory
Family 1	WT		No data	Family 1	WT		No
Family 2	WT		Yes, 2nd degree (1)	Family 2	WT		No
Family 3	WT		No	Family 3	WT		No
Family 4	WT		No	Family 4	WT		Yes, 2nd degree (1)
Family 5	BRCA1	p.A1708E	Yes, 1st degree (2)	Family 5	BRCA1	p.A1708E	No
Family 6	WT		No	Family 6	WT		No
Family 7	BRCA1		Yes, 2nd degree (1)	Family 7	WT	c.212+1G>A	Yes, 1st degree (1), 2nd degree (1)
Family 8	BRCA1	c.799delT	No data	Family 8	BRCA1	c.799delT	No
Family 9	WT		No	Family 9	WT		No

Supplemental Table 3. Recurrent mutations, among all patients in Guatemala and the US.

Recurrent mutation	Total	Guatemala	US
BRCA1 c.212+1G>A	18	18	0
BRCA1 c.798_799del	9	9	0
BRCA1 c.5123C>A	2	2	0
BRCA2 c.9235delG	2	2	0
BRCA2 c.2414delC	2	2	0
BRCA2 c.8363G>A	2	2	0
PALB2 c.3426_3429del	3	3	0
CHEK2 c.546C>A	2	2	0
BRCA2 c.3264dupT	2	0	2
BRCA1 c.68_69delAG*	1	0	1

* This mutation is an Ashkenazi Jewish founder mutation, known to be frequent in US Hispanic populations [23].

1.1 Appendix A: Medical and Family History Questionnaire

1. **Today's date**: __ __/__ __/__ __ __ __

2. Have you ever been diagnosed with breast cancer? Yes No

3. If yes, what is the month and year of diagnosis? __ __ / __ __ __ __

4. What is the month and Year you were born? (mm/yyyy) __ __ / __ __ __ __

5. How old were you when you had your first menstrual period? ________

6. Are you still having menstrual periods? Yes No

7. If not, how old were you when they stopped permanently? ________

15. What is your total yearly household income?

c Less than $15,000 (1) c $25,000-$35,000 (4) c $70,000-$90,000 (7)

c $15,000-$20,000 (2) c $35,000-$50,000 (5) c greater than $90,000 (8)

c $20,000-$25,000 (3) c $50,000-$70,000 (6) c Don't Know (9)

16. How many people are supported by this income? _________

17. What country were you born in? ____

18. What languages do you speak? c English

c Spanish

c Other ______________________

c Other ______________________

19. What is your current marital status?

c Married (1)

c Living with someone (4)

c Separated (2)

c Never Married (5)

c Divorced (3)

20. What is the highest level of education you have completed?

c Eighth grade or less (1)

c Bachelor's Degree (4)

c High School Diploma/GED (2)

c Master's Degree (5)

c Associate Degree (3)

c MD or PhD (6)

21. How many full sisters do you have, either living or deceased? _________

22. How many full brothers do you have, either living or deceased? _________

23. How many daughters and sons do you have? Do not include adopted, step, or foster children. **Daughters_______ Sons_______**

24. What are the birthplaces and languages of your parents and grandparents?

	**Country of Birth**	**First Language**
Father		
Mother		
Father’s father		
Father’s mother		
Mother’s father		
Mother’s mother		

25. Have you ever had a mammogram? Yes No

26. If yes, how old were you---- at the first? ______

27. How many mammograms have you had in the past 5 years?______

28. What is the month and year of your most recent mammogram? ___/_____

29. Have you ever had any of the following surgical procedures?

c Breast Biopsy

c Removal of Lump

c Total removal of breast

30. Have you had surgery to remove your uterus? Yes No

31. If yes, how old were you----? ______

32. Have you had surgery to remove one or both of your ovaries? c No c One c Both

33. *If yes, how old were you----? _______

40. In the diagram below, circle the family members that have had breast cancer. Or list family members in the spaces provided.

__________________________________ __________________________________

__________________________________ __________________________________

__________________________________ __________________________________

__________________________________ __________________________________

__________________________________ __________________________________
